# Concomitant Human Infections with 2 Cowpox Virus Strains in Related Cases, France, 2011

**DOI:** 10.3201/eid1912.130256

**Published:** 2013-12

**Authors:** Corinne Ducournau, Audrey Ferrier-Rembert, Olivier Ferraris, Aurélie Joffre, Anne-Laure Favier, Olivier Flusin, Dieter Van Cauteren, Kaci Kecir, Brigitte Auburtin, Serge Védy, Maël Bessaud, Christophe N. Peyrefitte

**Affiliations:** French Army Biomedical Research Institut, Grenoble and Lyon, France (C. Ducournau, A. Ferrier-Rembert, O. Ferraris, A. Joffre, A.-L. Favier, O. Flusin, C.N. Peyrefitte);; National Reference Center for Orthopoxviruses, Grenoble (C. Ducournau, O. Ferraris, C.N. Peyrefitte);; French Institute for Public Health Surveillance, Lyon (D. Van Cauteren);; Regional Hospital of Epinal, Epinal, France (K. Kecir, B. Auburtin);; Legouest Military Hospital, Metz, France (S. Védy);; UMR190 Aix Marseille University EHESP French School of Public Health, Marseille, France (M. Bessaud)

**Keywords:** cowpox virus, orthopoxvirus, rat-to-human infections, poxvirus, viruses, France, strains, co-infection, *Suggested citation for this article*: Ducournau C, Ferrier-Rembert A, Ferraris O, Joffre A, Favier A-L, Flusin O, et al. Concomitant human infections with 2 cowpox virus strains in related cases, France, 2011. Emerg Infect Dis [Internet]. 2013 Dec [*date cited*]. http://dx.doi.org/10.3201/eid1912.130256

## Abstract

We investigated 4 related human cases of cowpox virus infection reported in France during 2011. Three patients were infected by the same strain, probably transmitted by imported pet rats, and the fourth patient was infected by another strain. The 2 strains were genetically related to viruses previously isolated from humans with cowpox infection in Europe.

## The Study

**Figure 1:**
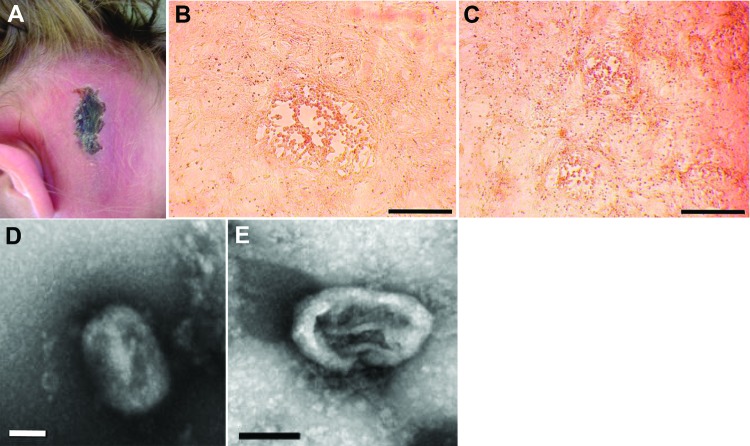
Cowpox virus infection in 4 persons in France. The case-patients were infected in 2011 by virus transmitted from infected pet rats. A) Cutaneous lesion on patient 1. B) Cytopathic effects observed on Vero cell monolayers with isolate CEPAD332. Scale bar represents 500 μm. C) Cytopathic effects observed on Vero cell monolayers with isolate CEPAD335. Scale bar represents 500 μm. D) Negative-staining electron microscopy image of isolate CEPAD332. Scale bar represents 100 nm. E) Negative-staining electron microscopy image of isolate CEPAD335. Scale bar represents 100 nm.

On September 12, 2011, an 8-year-old girl (patient 1) was admitted to the regional hospital of Epinal, France, for a cutaneous lesion on the lateral part of her neck that had evolved to a necro-ulcerative rash (Figure 1, panel A). Her sister (patient 2, age unknown) had similar cutaneous lesions. The family had purchased 4 rats (*Rattus norvegicus*) from a pet shop on August 19, 2011. The rats had been imported from a breeding facility in the Czech Republic by a local pet dealer. Locomotor disorders developed in 1 rat, and it died 4 days after the purchase. Two other rats became ill during the following weeks: the first, displaying symptoms of coryza, was examined by a veterinarian on September 5; the second was examined by the same veterinarian on September 12 for a vestibular syndrome that evolved to severe respiratory failure and then death on September 15. The fourth rat died without visible signs of disease. No biologic samples were collected.

Patient 3 was the 26-year-old female veterinarian who examined the sick rats. She kept their corpses in her office for a few days before destroying them. On September 20, she displayed cutaneous lesions similar to those of patients 1 and 2; samples of her lesions were collected 3 days later.

Patient 4 (age unknown) was the cousin of patients 1 and 2. He spent a few days in their house several weeks after the purchase of the rats but did not report any direct contact with them. On October 13, he displayed cutaneous lesions that were noticeably smaller than those of the other patients.

We conducted molecular testing of the patients’ lesion samples by using 14-kDa protein gene–targeting real-time PCR for orthopoxvirus detection (*1*). All samples gave a positive result with the orthopoxvirus probe and a negative result with the variola virus-specific probe, indicating the presence of orthopoxviruses with the exclusion of variola virus. The ≈600-nt PCR products were sequenced, and a BLAST search (http://blast.ncbi.nlm.nih.gov/Blast.cgi) identified cowpox virus (CPXV; family *Poxviridae*, genus *Orthopoxvirus*) in all cases.

The clinical samples were inoculated onto Vero cells (ATCC CC-81). Samples from patients 1, 2, and 3 gave rise to massive cytopathic effect (CPE), whereas no CPE was observed in cell cultures inoculated with samples from patient 4 (Table). Therefore, the samples from patient 4 were used to inoculate 2 additional cell lines, BHK-21 (ATCC CCL-10) and MRC-5 (ATCC CCL-171). A low CPE was observed 7 days postinoculation for both cell lines. An additional passage (passage 2) was performed on Vero cells for all the isolates. At this second passage, compared with the 3 other isolates, CEPAD335 (from patient 4) produced smaller plaques on Vero cells (Figure 1, panels B, C). Negative-stain electron microscopy performed on supernatant of cell cultures infected by isolates CEPAD332 and CEPAD335 showed typical poxvirus-like particles (Figure 1, panels D, E).

**Table:** Phenotypic properties of cowpox virus isolates from 4 patients, France, 2011*

Patient no.	Biologic samples in different cell lines			Isolate	Morphologic characteristics of isolates after passage 2 in Vero cells
	Vero	MRC5	BHK21		
1	High CPE	ND	ND	CEPAD332	High CPE on day 3, mean plaque size 600 μm ± 100 μm
2 (sister of patient 1)	High CPE	ND	ND	CEPAD336	High CPE on day 3, mean plaque size 600 μm ± 100 μm
3 (veterinarian)	High CPE	ND	ND	CEPAD333	High CPE on day 3, mean plaque size 600 μm ± 100 μm
4 (cousin of patients 1 and 2)	No CPE	Low CPE	Low CPE	CEPAD335	Low CPE on day 7, mean plaque size 300 μm ± 50 μm

*CPE, cytopathic effect; ND, not done.

**Figure 2:**
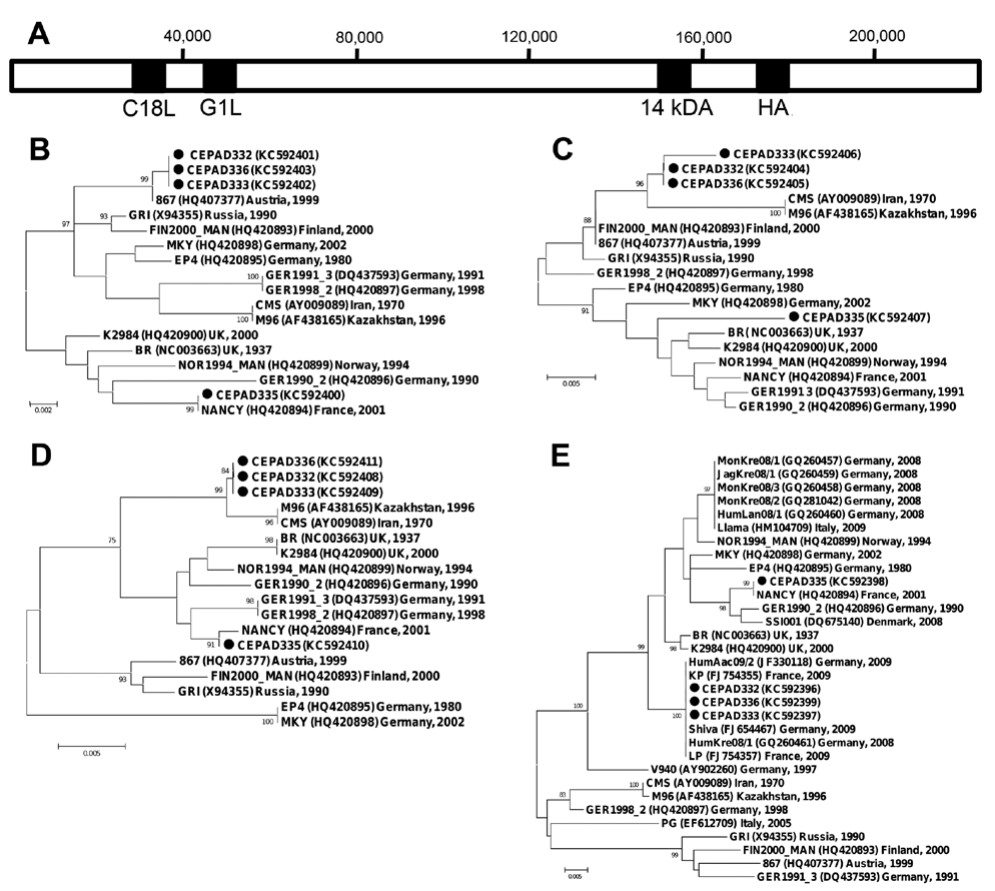
Schematic representation of cowpox virus (CPXV) GRI genome (A) and phylogenetic relationships between 4 genomic regions of CPXV isolates collected in France during 2011 and other CPXVs: C18L (B), 14-kDa (C), G1L (D), hemagglutinin (E). The sequenced regions are shaded in black in panel A. Nucleotide sequences were aligned by using CLC Main Workbench 6.0 software (CLC Bio,Aarhus, Denmark). Neighbor-joining phylograms were constructed in MEGA4 (www.megasoftware.net) by using the maximum composite likelihood method; the robustness of the resulting trees was assessed with 1,000 bootstrap replicates. The length of the branches is proportional to the number of base substitutions per site. The percentage bootstrap values are indicated if >70. Circles indicate the 2011 isolates from France; for other isolates, the year and country of isolation are indicated. Scale bars indicate nucleotide substitutions per site.

After DNA extraction from cell culture supernatants, the viral genome was amplified by PCR by using primer pairs that targeted 3 additional genomic regions (*2*,*3*): hemagglutinin (HA; ≈900 nt), C18L (≈850 nt), and G1L (≈850 nt), according to CPXV strain GRI nomenclature (Figure 2) (*4*). The sequences of the corresponding amplicons were determined and were deposited into GenBank under accession nos. KC592396–KC592411. The isolates CEPAD332, 333, and 336 were identical in the C18L, G1L, and HA regions; CEPAD333 diverged slightly from CEPAD332 and CEPAD 336 in the 14-kDa region (3 substitutions out of 576 nt). The results suggested that these 3 isolates originated from a unique CPVX strain. By contrast, CEPAD335 clearly diverged from the 3 others in all 4 studied regions (nucleotide divergence >2.5% in each region, large nucleotide insertions in the G1L region), indicating that patient 4 was infected by a different CPXV strain.

Phylogenetic studies were conducted by comparing the sequences of the 4 isolates with CPXV reference sequences (*4*–*6*) and sequences of CPXV isolates previously isolated in Europe (*7*–*11*) (Figure 2). In the HA region, CEPAD332, 333, and 336 showed 100% identity to several strains isolated in France and Germany in 2008 and 2009 from patients also infected by imported pet rats (*7*,*9*,*11*). Because these strains have not been sequenced in other genomic regions, it was impossible to study their relationships with our isolates.

In 3 genomic regions, CEPAD335 was closely related to isolate NANCY, a CPXV isolated in France in 2001 (*4*). Only 1 nt change was observed in each C18L and HA region; in G1L, CEPAD335 sequence contained a 84 nt-long insertion compared with NANCY, but only 1 nt change was observed in their matching parts. By contrast, CEPAD335 and NANCY were found relatively distant in the 14-kDa gene phylogram (18 nt changes out of 594 nt).

## Conclusions

At least 2 different CPVX strains were involved in the occurrence of 4 related human cases. Patients 1, 2, and 3 were probably infected by the same strain acquired from the pet rats, but the origin of the infection with the second strain remains unclear. We propose 2 hypotheses to explain these infections. The first hypothesis is the co-infection of the pet rat batch by the 2 CPXV strains. However, patient 4 did not report direct contact with the pet rats; furthermore, his lesions were observed at least 4 weeks after the death of the rats, while the CPXV incubation period is believed to be <2 weeks in humans (*12*). Alternatively, this patient might have been infected by other animals; he reported regular, close contact with horses and domestic cats that go outdoors, and horses and cats are known to transmit cowpox viruses to humans (*12*). In France and other European countries, human infection with CPXV has been known as a zoonosis transmitted mainly by feral cats and, more rarely, dairy cows. However, the outbreak of CPXV that occurred in Germany and France in 2008 in pet rat owners brought attention to this new source of infection. Several cases of pet rat–to–human CPXV transmission have been reported recently (*7*,*9*,*13*–*15*). The use of rodents as pets is likely to lead to an increase in CPXV human cases in the future, especially because persons younger than 30 years do not exhibit cross-reactive immunity conferred by smallpox vaccination, which was stopped at the end of the 1970s. 

Veterinary investigations were conducted in the pet shop in France where the 4 rats in this study had been purchased. Because no animals were ill, no samples were collected. No investigations were performed at the facility in the Czech Republic. Four previous human CPXV contaminations observed in France in 2009 were caused by contacts with infected pet rats that also originated from the Czech Republic (*9*).

Small wild rodents are believed to constitute the natural reservoir of CPXV, but this virus is able to infect a wide range of mammals, such as cats, cows, horses, elephants, and dogs (*12*). Our observation of human infections by 2 different CPXV strains genetically closely related to strains isolated years ago in France and Germany suggest the circulation of genetically stable viral strains among wild or domestic animals and their sporadic emergence among humans. Further studies regarding the molecular relationships between CPXV strains isolated from humans and from wild or domestic animals would help clarify the epidemiology of this virus.
